# Nearly 30 Years of Animal Models to Study Amyotrophic Lateral Sclerosis: A Historical Overview and Future Perspectives

**DOI:** 10.3390/ijms222212236

**Published:** 2021-11-12

**Authors:** Tiziana Bonifacino, Roberta Arianna Zerbo, Matilde Balbi, Carola Torazza, Giulia Frumento, Ernesto Fedele, Giambattista Bonanno, Marco Milanese

**Affiliations:** 1 Pharmacology and Toxicology Unit, Department of Pharmacy, University of Genoa, 16148 Genoa, Italy; bonifacino@difar.unige.it (T.B.); zerbo@difar.unige.it (R.A.Z.); balbi.phd@difar.unige.it (M.B.); torazza@difar.unige.it (C.T.); frumento@difar.unige.it (G.F.); bonanno@difar.unige.it (G.B.); milanese@difar.unige.it (M.M.); 2 Inter-University Center for the Promotion of the 3Rs Principles in Teaching & Research (Centro 3R), 56122 Genoa, Italy; 3 IRCCS Ospedale Policlinico San Martino, 16132 Genoa, Italy

**Keywords:** amyotrophic lateral sclerosis, genetic animal models, yeast, worm, fly, zebrafish, mouse, rat, guinea pig, dog, swine, non-human primates

## Abstract

Amyotrophic lateral sclerosis (ALS) is a fatal, multigenic, multifactorial, and non-cell autonomous neurodegenerative disease characterized by upper and lower motor neuron loss. Several genetic mutations lead to ALS development and many emerging gene mutations have been discovered in recent years. Over the decades since 1990, several animal models have been generated to study ALS pathology including both vertebrates and invertebrates such as yeast, worms, flies, zebrafish, mice, rats, guinea pigs, dogs, and non-human primates. Although these models show different peculiarities, they are all useful and complementary to dissect the pathological mechanisms at the basis of motor neuron degeneration and ALS progression, thus contributing to the development of new promising therapeutics. In this review, we describe the up to date and available ALS genetic animal models, classified by the different genetic mutations and divided per species, pointing out their features in modeling, the onset and progression of the pathology, as well as their specific pathological hallmarks. Moreover, we highlight similarities, differences, advantages, and limitations, aimed at helping the researcher to select the most appropriate experimental animal model, when designing a preclinical ALS study.

## 1. Introduction

Amyotrophic lateral sclerosis (ALS), also known as Charcot’s or Lou Gehrig’s disease, is a multisystem neurodegenerative disease, characterized by heterogeneity at the genetic, neuropathological, and clinical levels [1]. The progressive degeneration of upper and lower motoneurons (MNs) occurs during disease progression and affects pyramidal cells in the cortex, the corticospinal tract, and spinal motoneurons, usually sparing the extraocular and sphincter muscles [1,2,3]. The effects of MN loss evolve in muscle weakness, fasciculations, atrophy, spasticity, and hyperreflexia, eventually leading to paralysis, and patients typically die due to respiratory failure within three to five years from diagnosis [4,5]. Early pathogenic processes involve axonal degeneration and impairment of nerve terminal function, anticipating MN loss, and the onset of clinical symptoms [6].

Several causes have been proposed as the basis of the disease onset and progression, such as excessive calcium and glutamate excitotoxicity [7,8,9,10,11,12,13,14], oxidative stress [15,16,17], axonal damage and transport dysfunction [18,19,20,21], neuroinflammation [22,23], proteins misfolding and aggregation [24,25], proteasome impairment [26,27], endoplasmic reticulum stress [28,29], mitochondrial dysfunction and insufficient energy supply [30,31,32], and altered RNA processing [33,34]. Initially, ALS has been considered mostly as a MN disorder, but to date, much evidence confirms that ALS is a non-cell-autonomous disease, involving astrocytes, oligodendrocytes, microglia, and blood-derived immune cells [35,36,37]. Moreover, ALS forms a neurodegenerative disease continuum with frontotemporal dementia (FTD), and up to 50% of ALS patients concomitantly develop cognitive impairment or behavioral changes [38].

Most ALS cases are sporadic (sALS), and up to 10% have been classified as familial ALS (fALS) [39]. Currently, fALS-associated mutations have been found in approximately 50 genes, and more than 30 of them are thought to be causative [40,41]. The most common mutated genes are superoxide dismutase-1 (*SOD1*), chromosome 9 open reading frame 72 (*C9orf72*), fused in sarcoma (*FUS*), and TAR DNA-binding protein (*TARDBP*) [42]. Moreover, a significant fraction (about 20%) of sporadic cases carried a confirmed or likely pathogenic mutation, and almost all had no family history of ALS. This occurrence establishes the importance of routinely performing the genetic sequencing on ALS patients to improve disease subclassification, patient stratification, and clinical care [43].

In addition to genetic mutations, ALS may be also associated to environmental factors able to alter nerve cell functions [44].

## 2. Genes Involved in ALS

Mutations of the gene encoding for Cu/Zn SOD1 are among the most frequent ones in ALS [41]. Since SOD1 catalyzes the dismutation of superoxide anion in oxygen and hydrogen peroxide, the different mutations result in the decrease of free radicals detoxication (loss of function, LoF), but also in a toxic gain of function (GoF; [45]).

Mutations related the *TARDBP* gene, encoding the TAR DNA binding protein 43 (TDP-43), have been also identified in ALS [46]. Mutated TDP43 is indeed the main protein found in protein aggregates in the cytoplasm of MNs [47,48,49], contributing to the alteration of several cellular processes in ALS [50].

Other gene mutations involved in ALS are those related to the *FUS/TLS* gene, encoding for the RNA-binding protein FUS [51,52]. This protein is physiologically located in the nucleus but, when mutated, it aggregates in the cytoplasm of neurons [48,53], leading to cell death [49].

More recently, the repeated expression of hexanucleotide “GGGGCC” (G4C2) in the *C9orf72* gene’s non-coding region has been identified in ALS and demonstrated to alter critical cellular processes, including autophagy, membrane trafficking, immune response [54,55,56,57]. It is not yet clear why some patients with *C9orf72* repeat expansion manifest ALS phenotype, while others only FTD or both. Moreover, there is no apparent correlation between repeat size and disease severity [58]. To date, three different hypothetic mechanisms by which this genetic mutation may induce ALS have been hypothesized and none of them are exclusive [59]. The first hypothesis sustains that the repeat expansion may cause C9orf72 LoF, which contributes to neurodegeneration by yet unknown mechanisms. The second hypothesis is related to an RNA toxicity generated by G4C2 repeat-containing RNA foci that accumulate in the nucleus and induce the concomitant entrapment of other RNA-binding proteins that can no longer exert their physiological role. The third possibility deals with the accumulation of sense and antisense repeat proteins in the form of aggregation-prone dipeptide repeats.

All the above gene mutations contribute to ALS development and progression by triggering different toxic processes [60], such as oxidative damage and intracellular protein aggregates [17,61,62,63,64,65], axonal transport impairment [18], mitochondrial dysfunction [66], RNA metabolism impairment [67], and excitotoxicity [7,9,12]. We should also consider that genetic factors not only affect MNs, but also actively contribute to the degeneration or the activation of other CNS cells such as astrocytes and microglia, leading to neuroinflammation and other pathological phenomena [68].

Yet, there are other less frequent protein mutations involved in familial ALS form, such as: VAMP-associated protein B (VABP), Optineurin (OPTN), Valosin Containing Protein (VCP), Ubiquilin-2 (UBQLN2), Matrin 3 (MATR3), TANK-Binding Kinase-1 (TBK1), NIMA-related Kinase-1NEK1, and C21orf2 mutations [69,70].

Finally, it is important to consider also the interplay between environmental factors and genetic mutations that contributes to further complicate the multifactorial scenario of ALS [44,71].

## 3. Modelling Human ALS in Living Organisms

Following the initial discovery of mutations in fALS patients, it was possible to model the human disease in living organisms to elucidate the mechanisms underlying MN death. Even though the obvious limitations of animal models, and possibly for this very reason, their number is still growing in the attempt to obtain further items to identify adequate targets for the development of effective therapies, which this very complex disease urgently needs [72,73,74,75].

The purpose of this article is to provide a comprehensive and up-to-date overview of the available ALS animal models with the aim of offering details, including pros and cons, useful to design and optimize preclinical studies to investigate ALS. The present review is based on the collection of the most relevant articles present in the literature and focused on ALS animal models that have been identified on the PubMed^®^ online database by applying a systematic keyword cross-combination strategy, including genus/species coupled with ALS-linked genetic mutations.

In the following chapters, we will examine a variety of experimental models of the disease, from the most used to the least common, based on the ALS genetic mutations and the different organisms in which they have been reproduced. In addition, we have also included some very interesting non-genetic models in which MNs injury is based on autoimmune mechanisms or on the exposure to possible ALS environmental risk factors.

## 4. Rodent Models

Indeed, genetic mouse models have been playing a major role in elucidating the known pathways involved in the etiopathogenesis of ALS and in identifying new promising leads to further progress into clinical phases. The number of mouse models is still growing in parallel with the identification of new mutations, providing scientists with a variety of pathogenetic backgrounds and, even if each of them may present some drawbacks, they are an invaluable tool to increase our knowledge of ALS [74].

### 4.1. Rodents Carrying Cu/Zn Superoxide Dismutase 1 (SOD1) Mutations

After the discovery in the 1990s of the first ALS-linked gene mutation related to SOD1 [63], the first transgenic mouse was created that overexpressed the human protein with a glycine for alanine substitution in position 93 (SOD1^G93A^). Although developed on a specific point mutation that accounts for by 15–20% of the familial cases [76], this model is still the most studied and it has been instrumental to obtain important breakthroughs and in revealing the non-cell autonomous nature of ALS [73]. The *SOD1*^G93A^ mouse has been largely used for preclinical studies [77,78,79] and actively contributed to the introduction of riluzole and edaravone in ALS therapy [80,81].

This model clearly reproduces most of the pathological mechanisms of ALS in patients [82] and also recapitulates most of the motor deficits characterizing the human disease, showing a rapid degeneration of MNs that leads to paralysis and death within four to five months. Of great interest translational values, it also presents sex differences in disease progression [83,84].

Despite the clinical and mechanistic features that allow the researchers to study many aspects of the disease, a number of caveats has emerged that need to be considered for a correct use of this model in preclinical study design. The *SOD1*^G93A^ mouse model may spontaneously and progressively lose copy number of the mutation in the chromosome 21, which can directly impact the severity of the disease [74], thus acting as a confounding factor. Moreover, the background of the mouse strain and possible genetic drift can frequently affect the ALS phenotype, the pathology progression, and the life span, as confirmed by several pieces of evidence [85,86]. Of note, the overexpression of wild-type (WT) human SOD1 in mice has created quite a controversy since they exhibit axonopathies and other disease signs, although they appear delayed in respect to mutated transgenic mice [64]. Moreover, this observation could also challenge the role of the mutation as the determinant of neuronal injuries [87].

According to the currently known variants of *SOD1* mutations in ALS patients, numerous other models have been developed, including the *SOD1*^G37R^, *SOD1*^G86R^, and *SOD1*^G85R^ transgenic mice [61,88,89]. All these models express high level of mutated SOD1 and differs in onset, clinical aspects, progression of the pathology, and life span, albeit they share common traits, like early onset, micro/astrogliosis, neuroinflammation, glutamate excitotoxicity, mitochondrial alterations, defects in axonal transport, and MN loss leading to progressive paralysis of hind limbs and finally death [73].

Mutated *SOD1* rat models have also been developed, particularly with G93A and H46R mutations, showing genetic alteration-derived pathological features similar to those described in mice. They showed upper and lower MN degeneration and the disease severity was directly proportional to mutant *SOD1* copy expression [90,91]. In particular, the G93A mutation caused a more aggressive disease than the H46R one [92]. Rat models offer the advantage of a greater size facilitating handling for behavior and in-vivo treatment procedures such as intrathecal catheters application, ICV administration, implant of osmotic pumps for chronic drug treatments with minimal interference to performances in behavioral tasks, and more availability of tissues for ex-vivo analyses. However, rats imply higher maintenance costs and logistic issues, including availability of space and greater drug needs for chronic treatments.

### 4.2. Rodents Carrying Tar DNA-Binding Protein 43 (TDP-43) Mutation

Other important mutations have been found in the gene encoding for TDP-43 (*TARDBP*) that accounts for approximately 4% of FALS patients and are also present in several apparent SALS cases [47]. Furthermore, mutated TDP-43 is the main component of ubiquitinated inclusions in most cases of ALS [93]. Therefore, mouse and rat carrying *TARDBP* mutations represent other important models to further investigate neurotoxic mechanisms in ALS.

At present, approximately 20 TDP-43 mouse models have been established since the discovery of these mutations in fALS [74]. Although the development of this model was very challenging as the disease severity with TDP-43 mutants is dose-sensitive, *TARDBP* silencing can lead to neurodegeneration and minimal overexpression of wild-type (WT) or mutated TDP-43 may cause multiple RNA changes and nonspecific toxicity [94]. Moreover, TDP-43 mutants seem to have diet-related effects on phenotype [73,74].

The first transgenic TDP-43 mouse models overexpressed wild type or mutant (A315T and M337V) TDP-43 cDNAS and were obtained by using a prion protein (Prnp) gene promoter [95,96,97]. These transgenic mice accumulated pathologic aggregates of ubiquitinated proteins in specific neuronal populations, leading to a severe phenotype characterized by neuronal morphological abnormalities, cytoplasmatic inclusions, and varying degrees of spinal cord pathology. As said above, phenotype severity is correlated with levels of mutated TDP-43 expression and death occur within the first weeks of life in those animals with high expression. However, TDP-43 aggregates were rare or absent in these models [95,96,98]. Moreover, overexpression of A315T, G298S, and even WT TDP-43, obtained by using Thy1 promoter, trigger a pathological phenotype [99,100].

Later, Prnp promoter was further engineered to express wild type and other mutant TDP-43 cDNAs, resulting in a less aggressive phenotype [101]. In particular, overexpression of M337V, Q331K, and WT TDP-43 induced motor impairment in three-month-old mice leading to tremor, hind limb coordination, and muscle strength deficits. The Q331K mutation is the less aggressive, showing EMG recording impairments at ten months with reduced MN loss, non-progressive axonal degeneration, and reduced NMJ damage. In addition, TDP-43 cytosolic aggregations or nuclear export were no present, but rather a significant deficit in systemic RNA splicing and regulation [73,74,101].

On the contrary, in TDP-43-A315T transgenic mouse model, ubiquitinated TDP-43 cytoplasmic inclusions have been described [102]. Of note, in this latter case only lower MNs are involved, and the progressive degeneration stops at 20 months without causing lethality. Importantly, Mitchell and collaborators (2015) have demonstrated that the concomitant expression of Prnp-Q311K and wild type alleles developed a more aggressive phenotype with MN loss, TDP-43 inclusions, and reactive gliosis leading to death in eight to ten weeks [103]. In conclusion, TDP-43-Q331K mice showed convincing ALS phenotypic features, but also problematic construct validity based on strain and promoter-dependent effect [104].

New additional strategies have been developed in generating TDP-43 transgenic mice. One of these includes the incorporation of fragments from human genomic BAC containing libraries, taking advantage of the endogenous TDP-43 promoter to produce transgenic A315T, G348C, and wild type mice [105]. These mice showed TDP-43 overexpression in spinal cord, motor deficits, and age-related neuroinflammation from 42 weeks of age, deficits in spatial learning at around one year, nuclear and cytosolic ubiquitinated TDP-43 inclusions at ten months, but normal lifespan [105].

Another model expresses a form of TDP-43 that misses the nuclear localization signal (NLS) in a physiological fashion. This model was used in combination with calcium/calmodulin-dependent protein kinase II alpha promoter to drive doxycycline expression in neuronal cells, showing accumulation of cytosolic TDP-43 with minor aggregates, without developing an ALS phenotype [106]. The subsequent modification of this line, by using the neurofilament heavy chain (NEFH) promoter to regulate doxycycline suppression, induced a rapid and robust neurodegeneration including MN loss, denervation of NMJ, TDP-43 mislocalization, motor deficits, and premature death. All these symptoms were partially reversible upon doxycycline administration [107].

Novel mouse lines with point mutations within the endogenous *TARDBP* gene were used to dissect the molecular effect of TDP-43 LoF or GoF in vivo in the absence of a transgene. In this context, the p.M323K mutation in the C-terminal low complexity glycine rich domain of TDP-43 caused splicing GoF associated to the development of late progressive neuromuscular phenotype with partial MN loss. On the contrary, the p.F210I mutation, leading to a LoF in the RNA binding activity of TDP-43, did not cause any pathological phenotype [108].

In general, we can assume that TDP-43 GoF in the cytoplasm is likely implicated in the mechanisms of ALS-associated TDP-43 mutations, but it should be also considered the clear heterogeneity of the phenotype that depend on the expression levels of the mutant protein and/or the genetic background of the animal model (mPrp vs. mTardbp promoter; [109]).

Next to transgenic mice, both WT and M337V TDP-43 have been overexpressed also in rats [110]. Expression of M337V TDP-43 from the human TDP-43 promoter caused early mobility problems, paralysis, and death before sexual maturity. Interestingly, rats expressing comparable levels of WT TDP-43 did not develop paralysis by 200 days, suggesting that mutant TDP-43 is more toxic than WT protein.

### 4.3. Rodents Carrying the RNA-Binding Protein Fused in Sarcoma (FUS) Mutations

Like TDP-43, FUS is an RNA- and DNA-binding protein involved in ALS pathophysiology that plays a role in numerous cellular processes, including transcription, splicing, microRNA maturation, RNA transport, and stress granule formation [111]. The presence of cytoplasmic FUS protein aggregates and the subsequent nuclear depletion is an important hallmark that contribute to ALS pathogenesis [67]. Since its identification [51,53], several mouse models carrying FUS mutations have been developed, most of which are responsible of NLS disruption [112]. It has been shown that loss of FUS does not result in ALS phenotype [113], supporting the mutant FUS GoF as fundamental to develop the disease [114,115]. Usually, transgenic FUS mice present protein aggregation in MNs, leading to NMJ damage [114], inhibition of protein transport between endoplasmic reticulum and Golgi complex in neuronal cells [116], and cell death [117].

Homozygous *FUS* mice showed high lethality paralleled by apoptotic MNs and cytosolic FUS mislocalization, whereas heterozygous ones presented increased cytoplasmic, but not nuclear, FUS level, and progressive MN loss (approx. 30% at 22 months of age) without proceeding to the end stage of the disease [115].

Other transgenic lines were produced, including the insertion of both the wildtype and multiple mutant FUS-containing cDNAs in the microtubule-associated protein tau (Mapt) gene locus. The models expressing this mutant form displayed NMJ deficits, MN loss, cytoplasmic FUS mislocalization, and aggregation, but no paralysis and death [114].

A humanized knock-in model recapitulating a patient 3′splicing gene defect was developed a few years ago [118]. These *FUSDelta14* heterozygous mice manifested progressive altered motor functions at 12 and 15 months of age, hind limb muscle denervation at 18 months, paralleled by MN loss, and reduced lifespan at 22 months. Moreover, this model showed increased FUS cytosolic localization with no apparent aggregates.

Other transgenic mice with a more aggressive ALS phenotype originated from FUS overexpression using promoters such as Prnp or Thy1 genes. The use of Thy1 promoter to drive FUS cDNA with mutations in NLS guided to hemizygous mice with a rapid motor phenotype (2.5–4.5 months) leading to death a few days after the onset. These mice showed neuroinflammation and presented FUS inclusions in MNs and in other neuronal cells [119]. Moreover, the use of Prnp promoter to induce the expression of human wild type FUS cDNA was incompatible with the survival of different founder lines. Homozygous mice showed cytosolic FUS inclusions, neuroinflammation, tremor, and hind limb dysfunction at four weeks, with death occurring at 10–13 weeks [120]. Another mouse model was generated by using Prnp promoter to insert the *FUS*^R521C^ mutation and showed severe motor deficit, cytosolic FUS inclusions, and premature death [121]. In this model, a genetic background effect has been also identified, with survival varying from about 50–70 to 130–150 days of age.

Transgenic rats have been also produced by intravenous administration of adeno-associated virus (AAV9) FUS. These rats showed progressive motor alteration and respiratory dysfunction [122]. Overexpression of R512C mutant human *FUS* in rats induces motor axon degeneration with progressive paralysis, neuron loss in cortex and hippocampus, protein aggregation, and glial reactivity at early ages. Of note, transgenic rats overexpressing wild type human *FUS* were asymptomatic at the beginning of life, but they showed deficit in spatial learning and memory as well as significant loss of cortical and hippocampal neurons at advanced ages (12 months) [123]. As for mice, overexpression of mutant *FUS* seems to be more toxic than WT *FUS*.

### 4.4. Rodents Carrying Chromosome 9 Open Reading Frame 72 (C9orf72) Mutations

In addition to *SOD1* and RNA-binding protein mutations, genetic studies identified the location of the *C9orf72* gene in the chromosome 9p21 locus in which mutations are linked to the GGGGCC repeated expansion (G4C2) [54,58,124,125]. In affected patients, such sequence was found expanded from hundreds to thousands of repeats in the gene and is the most common cause of familial ALS and FTD, thereafter called C9ALS-FTD [54].

Although all the functions of the C9orf72 protein are still a matter of investigation, several lines of evidence indicate a key role in the modulation of autophagy, endo-lysosomal trafficking, and immune response [126].

The first mouse model carrying a *C9orf72* gene mutation was developed in 2013 by Xu and collaborators [127]. These mice presented NMJ damage, hippocampal cell disorganization, cognitive impairments, apoptosis, and gait deficits [128]. In addition, they also showed protein inclusions of TDP-43 and di-peptides, loss of Purkinje and cortical neurons, astrogliosis, weight loss, hyperactivity, and motor deficits [129,130,131].

*C9orf72* knock-out mice have been created to evaluate the LoF effects [132,133,134,135]. In this view, a conditional KO mouse with a neuronal specific loss of C9orf72 has been generated that, however, did not manifest MN degeneration, frank motor deficits, or decreased survival [132]. Knock out mice were also produced by the Knock-Out Mouse Program (KOMP) and the zinc finger nuclease (ZFN)-mediated genome editing and they displayed splenomegaly and lymphadenopathy, with engorged macrophage-like cell accumulation [133,135] and age-related neuroinflammation, like that observed in *C9orf72*-related ALS patients [133]. Moreover, in the *C9orf72* KOMP mouse model, a significant decrease in survival was also observed, occurring at around 100 days of age [136]. Finally, the creation of a third type of knock-out mouse model pointed out a robust immune phenotype, including myeloid expansion, T cell activation, and increased plasma cells, leading to an elevated level of autoantibodies and immune-mediated glomerulonephritis [137]. In general, these models did not confirm the role of C9orf72 LoF in ALS, but highlighted that the therapeutic approaches aimed at reducing this protein could trigger possible autoimmune consequences.

Later, four transgenic mouse models expressing the BAC human *C9orf72* repeat expansion have been generated [134,138,139,140]. These models shared similar features, with some peculiar differences. All of them developed the *C9orf72* ALS hallmarks, such as RNA foci in the nucleus and cytoplasmatic repeat-associated non-ATG (RAN) translation inclusions, even if differences in the expression level were observed [138]. Interestingly, only the transgenic model developed by Liu and collaborators (2016) displayed widespread nuclear and cytoplasmic TDP-43 inclusions in neurons, MN loss, muscle denervation, hind limb paralysis, and decreased survival [130]. A possible explanation of this peculiar aspect might lay on the different genetic background, since the FVB/NJ background was used in the latter model while C57BL/6J was that of the former.

In conclusion, the *C9orf72* mouse models are useful as preclinical models for new therapeutic research both in-vitro and in-vivo [140,141], but depending on the experimental design of the study and on the disease outcome to be monitored, it is crucial to consider the different pathological traits that characterize the above-described mouse models.

With regards to rats, it has been reported that ablation of the *C9orf72* gene, using the CRISPR/cas9 technique, produced only some alterations to cervical lymph nodes and spleen, but no MN loss, thus supporting the notion that C9orf72 LoF alone is not responsible for ALS-like degeneration [142]. Interestingly enough, when these *C9orf72* KO rats were treated with low doses of kainic acid for four weeks, they developed significant MN death that was accompanied by progressive motor deficits, suggesting that C9orf72 LoF could trigger sensitization of MN to other risk factors, such as excitotoxicity, to induce ALS.

Recently, *C9orf72* knock-in rats were generated by knocking in 80 G4C2 repeats with human flanking fragments within exon1a and exon1b at the rat *C9orf72* locus. These rat models reduced C9orf72 protein expression in several CNS areas and showed motor deficits from four months of age due to motor neuron loss, thus being a further useful model for investigating the contributions of loss-of-function to neurotoxicity in *C9orf72*-related ALS [143].

## 5. Rodent Models Carrying Rare Mutations Linked to ALS

### 5.1. Rodents Carrying Alsin Mutations

To date, two loci generating autosomal recessive forms of ALS have been linked to mutations in the *ALS2* gene, localized to chromosome 2q33 [144,145]. This ubiquitously expressed gene, containing three different guanine-nucleotide-exchange factor-like domains, encodes for the 184-kDa protein alsin, which may play a role in the etiology of the disease [144,145]. Several *ALS2* gene mutations have been identified, but only four (261delA, 553delA, G660A, and 1130delAT) were found in the RCC1 domain region expected to affect the protein, with the two mutations nearest to the amino-terminus (261delA and 553delA) resulting in frame shift mutations responsible for the development of juvenile ALS termed ALS type 2 (ALS2) [144,145,146].

As reviewed by Chandran et al. (2007) [147] and Cai et al. (2008) [148], to date four *ALS2*-deficient mouse models have been developed [149,150,151,152] to shed light on the impact of alsin deficiency in ALS, but they largely failed to recapitulate hallmarks of motor neuron disease [149,150,151,153].

### 5.2. Rodents Carrying Senataxin Mutations

Senataxin (SEXT) is an RNA-binding protein with a highly conserved helicase domain, but it does not possess a low-complexity domain, making it unique among ALS-linked disease proteins. The mutation of the *SEXT* gene is the cause of a juvenile form of ALS termed ALS type 4 (ALS4). In 2018, Craig and collaborators introduced the first mouse model carrying *SEXT* gene mutations and causing ALS4 [154]. To date, the mechanistic basis for motor neuron toxicity is still unknown. ALS4 has been modeled in mice by expressing two different *SEXT* gene mutations (R2136H and L389S) via transgenesis and knock-in gene targeting, respectively. Both mouse models developed neuromuscular phenotypes and motor neuron degeneration, clearing of TDP-43 accompanied by TDP-43 cytosolic mislocalization that are consistent with the pathologic hallmarks of the human disease. Nuclear membrane abnormalities, related to nucleocytoplasmic transport proteins Ran and RanGAP1 in MNs, have been disclosed in *SETX* ALS4 mice and nuclear import was delayed in *SETX* ALS4 cortical neurons, indicative of impaired nucleocytoplasmic trafficking. Thus, *SETX* ALS4 mice recapitulate ALS disease phenotypes in association with TDP-43 mislocalization and provide insights for TDP-43 pathology, linking SETX dysfunction to common pathways of ALS MN degeneration.

### 5.3. Rodents Carrying Optineurin Mutations

Optineurin (OPTN) is a protein mainly implicated in autophagy processes. *OPTN* gene mutations have been linked to the pathogenesis of both familial and sporadic ALS cases [155,156,157]. Specifically, different *OPTN* gene point mutations have been identified as being causative of primary open-angle glaucoma in patients with ALS: a homozygous deletion of exon 5, a missense mutations R96L, a homozygous Q398X nonsense mutation, and a heterozygous E478G missense mutation [158,159]. Moreover, a transgenic knock-in mouse was generated replacing wild-type *OPTN* by the *OPTN*^D477N^ mutant, equivalent to the *OPTN*^D474N^ as non-disease-related human mutation of *OPTN*, and no motor phenotype alterations were registered [160].

### 5.4. Rodents Carrying Ubiquilin-2 Mutations

Ubiquilin-2 (UBQLN2) plays a central role in the ubiquitin proteasome system (UPS) and its dysfunction results in protein aggregation [161]. Several *UBQLN2* gene mutations have been linked to ALS and FTD as reviewed by Renaud and collaborators in 2019 [162], and many transgenic *UBQLN2* rodent models have been developed. Transgenic mouse models expressing h*UBQLN2*^P497H^ manifested cognitive deficits, dendritic spinopathy, and UBQLN2 inclusions in the hippocampus, but neither TDP-43 pathology nor loss of motor neurons. Similarly, a rat model carrying the same mutation displayed cognitive deficits associated with UBQLN2 aggregates in hippocampus and evidence of neuronal death [163,164]. Knocked-in h*UBQLN2*^P506T^ mice were also originated, showing cognitive impairment, but again, no motor deficits [165]; whereas mice carrying the h*UBQLN2*^P497S^ or h*UBQLN2*^P506T^ mutations exhibited MN loss and cognitive impairments [166].

Interestingly, a double transgenic mouse model harboring both h*UBQLN2*^P497H^ and h*TDP-43*^G348C^ mutations showed MN loss and muscle atrophy associated to motor and cognitive deficits during aging [167].

### 5.5. Rodents Carrying Profilin 1 Mutations

Profilin 1 (PNF1) is a protein encoded by the *PFN1* gene and it is known to play an important role in cytoskeletal structure by regulating actin filament formation, driving cell motility and other actin-linked processes [168,169]. Several *PFN1* gene mutations are linked to ALS (C71G, M114T, E117G, G118V) [170,171], but how these mutations can result in ALS is still not well understood. Both LoF and GoF mechanisms have been proposed to take place. *PFN1* gene mutations accelerated the protein turnover in cells [172], altered microtubule dynamics by affecting the growth rate of microtubules and leading to MN degeneration [173], increased dendritic arborization and spine formation, and induced cytoplasmic inclusions [174].

Hemizygous *PFN1*^G118V^ transgenic mice exhibited many pathological features of ALS, including loss of lower and upper MNs, loss of MNJs, aggregation of the mutant profilin 1 protein, abnormally ubiquitinated proteins, increase in nuclear staining of phosphorylated TDP-43 in the spinal cord, fragmented mitochondria, glial cell activation, muscle atrophy, weight loss, and reduced survival [175]. Motor dysfunctions occurred at 120–130 days and the end-stage of the disease was around 165–210 days of life.

Expression of *PFN1*^C71G^ mutation has been induced in mice showing a progressing phenotype [176]. The hemizygous mice showed slight weakness at 350 days, while the homozygous anticipates the onset of the phenotype (150 days) and full paralysis (320 days). Other transgenic mice were attained by breeding a Prnp-driven *PFN1* transgenic line in hemizygous state with a Thy1-*PFN*^1C71G^ homozygous line, obtaining an accelerated ALS pathology [176]. Recently, Brettle and collaborators [177] developed a novel mouse model expressing *PNF1*^C71G^ under the control of the Hb9 promoter, targeting the mutation to α-MNs in the spinal cord during development. Adult mice presented significant motor deficits and a strong reduction in the number of MNs in the spinal cord, thus supporting the effect of profilin 1 during neurodevelopment.

### 5.6. Rodents Carrying Valosin Containing Protein Mutations

Valosin containing protein (VCP) is a protein involved in ER biogenesis and maintenance, whose gene mutations have been linked to several neurological disorders, including ALS [178,179,180]. R155H or A232E mutations have been introduced in the *VCP* gene to obtain murine VCP-related disease models [181,182]. The heterozygous knock-in VCP^R155H^ mouse model exhibited pathological characteristics of the human disease in muscles, bones, and brain, including age-dependent degeneration of ventral horn MNs, TDP-43-positive cytosolic inclusions, mitochondrial abnormalities, and progressive astrogliosis. Although these animals do not develop rapidly a progressive and fatal ALS-like pathology, they do recapitulate some key pathological features of the disease [183,184]. The homozygous *VCP*^R155H^ mouse showed muscle, bone, and brain alterations like those of the heterozygous model, but at a very early stage, and mice usually survive less than one month [185].

### 5.7. Rodents Carrying Vesicle-Associated Membrane Protein (VAMP)-Associated Protein B Mutations

VAMP-associated protein B (VAPB) belongs to the VAMP-associated proteins that play important functions in membrane trafficking [186], neurotransmitter release, and lipid transport [187]. They also participate in the unfolded protein response machine, an ER activity that suppresses accumulation of misfolded proteins to maintain cell viability and function [188]. VAPB has been connected to various neurodegenerative diseases, including ALS [189,190]. Indeed, several mutations of the *VAPB* gene have been observed in patients with ALS, such as P56S, T46I, del160, D130E, A145V, S160Δ, and V234I [58,191,192,193]. Transgenic mice overexpressing the WT or the P56S mutant human VAPB protein, with either neuron-specific or ubiquitous promoters, have been generated [194,195,196], but only animals expressing mutant *VAPB*^P56S^ showed motor behavior defects [195]. Later, Larroquette and collaborators [197] derived and characterized knock-in *VAPB* mice with the P56S mutation. These mice exhibited late-onset motor behavior defects, characteristic of patients carrying this mutation, and displayed many cellular pathological features implicated in ALS (e.g., accumulation of ubiquitinated proteins, ER stress, and autophagic response) before motor behavior defects.

## 6. Non-Genetic Rodent Models

### 6.1. Guinea Pig ALS Models

The first non-genetic model of ALS has been created in guinea pigs by using an ascorbic acid deficient diet inducing MN degeneration, muscle atrophy, and demyelination of the pyramidal tracts [198]. In most of the studies, guinea pigs have been exploited to focus on the autoimmune etiology of ALS. In this view, two models were set up by injections of purified bovine spinal MN antigen or of bovine ventral spinal cord homogenates in complete Freund’s adjuvant. In the first model, named “experimental autoimmune motor neuron disease”, animals displayed loss of lower MNs, neuromuscular degeneration, and limb weakness [199]. The second model, known as “experimental autoimmune gray matter disease”, showed the involvement of both upper and lower MNs, with denervation occurring in the motor cortex and spinal cord [200]. Successively, another model was developed and characterized by guinea pig immunization with choline acetyltransferase purified from human placenta. Animals showed lower MN degeneration, muscle atrophy, and a gradual weakness, mainly at the hind limb [201].

### 6.2. L-BMAA-Induced ALS Rodent Models

Besides genetic alterations, also environmental factors are known to play a role in ALS development [202,203] and, therefore, non-genetic rodent models have been generated. Among the environmental risk factors, exposure to beta-N-methylamino-L-alanine (L-BMAA), a nonprotein amino acid present in cycad seeds and produced also by different taxa of cyanobacteria, has been strongly associated to ALS-PDC (Parkinson’s Disease Complex) syndrome, also known as Guam disease given its characterization in the Chamorros population of the Guam island [71].

In a first study, 21-day-old rats were i.p. injected with different doses of L-BMAA (up to 350 mg/kg/die) for five days and then analyzed for up to eight months with a set of neurological and functional tests [204,205]. At doses greater than or equal to 200 mg/kg, animals displayed significant time-dependent neurological and functional deficits as assessed with ambulation, tail lift and strength tests, rotarod performance, and footprints analysis. In addition, electron microscopy, immunohistochemical, MRI, and Western blot analyses indicated the presence of damaged motor neurons, increase of high molecular forms of TDP-43 in the lumbar spinal cord and motor cortex, decreased motor cortex and muscle volume, alteration of bulbar nuclei.

A second model was developed by intrathecal infusion of L-BMAA (5 mM) to adult rats for 30 days via catheter-connected mini-osmotic pumps [206]. This chronic model showed a spectrum of degenerative alterations (e.g., MNs shrinkage and death, TDP-43 cytosolic appearance, GLT-1 reduction, reactive astrogliosis, microglia infiltration) in the ventral horn similar to that observed in age-matched SOD1^G93A^ mutant rats; however, no behavioral analysis was reported.

Finally, also i.v. infusion of L-BMAA (300 mg/kg) for three consecutive days in adult rats resulted in a model that resembled many traits of ALS [207]. Indeed, treated animals showed significant time-dependent motor deficits, as well as mitochondrial degeneration, reduction of GLT-1 levels, loss of nuclear and appearance of cytosolic TDP-43 in degenerating MNS, neuronal death, reactive gliosis.

Although the L-BMAA model also shows features of other neurodegenerative diseases, it may represent a fruitful additional tool for ALS-related mechanistic and therapeutic studies.

## 7. Drosophila Melanogaster (Fruit Fly) Models

*Drosophila melanogaster* is an animal model very useful to study neurodegenerative disease, including ALS [208,209], since it is easy to handle, cost-effective, with short life cycle and complete genome sequence [210,211]. Another important feature that makes *Drosophila melanogaster* a powerful genetic model is the UAS/Gal4 system [212], which is extensively used to overexpress both *Drosophila melanogaster* and disease-associated human genes. Like zebrafish, about 77% of the human ALS-associated genes have fly orthologs [213]. To date, several different ALS-linked mutations have been expressed in *Drosophila melanogaster* [214,215]. On these premises*, Drosophila* is considered a suitable and alternative model for studying genetic issues in many neurodegenerative disorders, including ALS [216,217,218,219,220].

### 7.1. Drosophila Melanogaster Carrying SOD1 Mutations

Several studies have shown that inactivating the *Drosophila melanogaster* SOD1 enzymatic activity by deletions or missense mutations of the enzyme led to several pathological phenotypes. In the fly model of SOD1 LoF, lifespan was drastically reduced by 85–90% and locomotor activity was also impaired. In addition, the resistance to oxidative stress conditions was lowered and fertility and wing morphology were abnormal [221,222,223]. In the fly SOD1 LoF model, the expression of WT human SOD1 fully rescued the lifespan reduction, whereas the expression of different fALS-related human SOD1 mutants (*SOD1*^A4V^, *SOD1*^G37R^, *SOD1*^G93C^, *SOD1*^G41D^, *SOD1*^I113T^) resulted in a partial rescue, only. The lifespan reduction was paralleled by an early drop of negative geotaxis performance in line with the pathological phenotypes [224].

Fly models were also generated using the UAS/Gal4 system [212] to overexpress different human *SOD1* transgenes directly in MNs. The UAS/Gal4-driven expression of either WT or ALS-related SOD1 forms (A4V or G85R) in MNs did not alter lifespan in flies [225], but induced progressive motor function deterioration. Since the different phenotypes observed depended not only on the transgene expression level but also on the cellular type targeted [225,226], a new model has been generated by introducing ALS-related human *SOD1* mutations at the conserved residues in fly SOD1, thereby creating fly *SOD1*^G85R^, *SOD1*^H71Y^, and *SOD1*^H48R^ mutants [227]. In homozygous condition, these mutants died during development, with escaper adult flies showing shortened lifespan, severe motor defects (even in the absence of MN death), and reduced number of NMJ boutons [228]. In general, we can summarize that fly models carrying *SOD1* mutations may display different pathological features depending on the specific mutated gene, spanning motor deficits, focal accumulation of SOD1 in MNs, glial cell enlargement [225], locomotor disturbances, neuronal degeneration, muscle retraction, reduced survival [227], and mitochondrial dysfunction [229]. On this basis, future studies using *Drosophila melanogaster* could help to understand the mode of propagation of misfolded SOD1, as well as the physiopathological relationships between MNs, glial cells, and muscles.

### 7.2. Drosophila Melanogaster Carrying TDP-43 Mutations

Since TDP-43 is highly conserved during evolution [230], it makes fly an ideal organism to study its function. TBPH TAR DNA-binding protein-43 homolog (*TBPH*) is the fly ortholog of *TARDBP* gene and LoF and GoF approaches have been modeled in fly to unravel TDP-43 functions.

In *TBPH*-null mutant flies, mortality was observed at the second instar larval stage [231], at the pupal stage [232], or during development, including a few adult progeny [233,234,235,236]. These *TBPH* gene mutants displayed eclosion, climbing, and crawling defects, decreased or increased synaptic bouton number, reduced dendritic branching, impaired synaptic transmission, and axonal transport dysfunctions [233,234,235,236,237,238,239]. High levels of *TBPH* were very toxic, causing premature mortality [232,235,236,238,240], reduced lifespan [235,241,242], larval locomotion defects [235], and age-dependent climbing deficit [243,244].

*Drosophila melanogaster* was also used to assess the effects of several human TDP-43 mutations. In this respect, both TDP-43^G294A^ or TDP-43^M337V^ homozygous flies had normal development and lifespan, the larval locomotion was unaltered, and their climbing capacity was only slightly reduced in respect to age-matched controls [245,246]. On the contrary, overexpression of other TDP-43 ALS-linked mutations led to progressive degeneration and several functional deficits in flies depending on the single mutation at issue, like aberrant eye morphology (D169G, G298S, A315T, M337V, N345K), reduced life span and eclosion defect (G287S, A315T, G348C, A382T, N390D), climbing and crawling deficits (A315T), increased larval turning time, reduced bouton numbers, increased or no change of dendritic branching, increased active zone at NMJs (D169G, G298S, A315T, N345K), day and night sleep fragmentation (D169G, G298S, A315T, Q331K, N345K), cytoplasmic and axonal aggregates (D169G, G298S, A315T, N345K) [241,247,248,249,250]. In other cases, mutant phenotypes cannot be clearly distinguished from those carrying WT TDP-43, since the latter shows similar characteristics to the mutant forms; in fact, also the overexpression of the human WT TDP-43 may cause aberrant eye morphology, eclosion defect, climbing and crawling defect, learning deficiency, vesicle transport dysfunction, increased or no change of dendritic branching at NMJs, and TBPH aggregates [233,235,237,239,247]. The above-mentioned controversial results among the different mutant lines may be due to differences of the genetic backgrounds and/or of the position of the UAS-*TBPH* insertion. Moreover, overexpression of mutant *TBPH* (A315T, Q367X) was reported to induce aberrant eye morphology, axonal aggregates, crawling defect, eclosion defect, decreased bouton numbers, and decreased or no change in dendritic branching at NMJs [236,247].

### 7.3. Drosophila Melanogaster Carrying FUS Mutations

The cabeza (*caz*) is the only ortholog gene of FUS in *Drosophila melanogaster* [251]. Caz LoF models, obtained by knockout strategy, showed aberrant eye morphology, decreased viability, life span reduction, crawling and climbing defects, reduced synaptic branches and bouton number [237,252,253,254,255,256]. GoF models, by GAL4/UAS-induced overexpression of WT or mutated human *FUS* have been also investigated. The overexpression of human *FUS* mutants led to several phenotypic alterations, including aberrant eye morphology (R518K, R521C, R521G, R521H, R524S, P525L), eclosion defect (R518K, R521C, R521H, R521G), climbing and crawling defects (R518K, R521C, R521G, R521H, R524S, P525L), reduced synaptic bouton number (R521G, R524S, P525L) or no change (R521C, R521H), and decreased active zone at NMJs (R518K, R521C, R521H, P525L) [237,256,257,258,259,260], indicating that either the protein LoF or Gof induced a pathological phenotype.

As in the case of TDP-43, the overexpression of WT human FUS induced a phenotype characterized by alterations that were almost completely superimposable to those observed with the different *FUS* mutants [237,256,257,258,259,261].

### 7.4. Drosophila Melanogaster Carrying C9orf72 Mutations

*Drosophila melanogaster* does not have a *C9orf72* ortholog gene [262], thus making it impossible to determine the consequence of C9orf72 LoF in this organism. Therefore, fly models for *C9orf72*-associated ALS have been developed by overexpressing G4C2 repeat RNA, modelling DPR protein toxicity, thus revealing important molecular insights [263]. Transgenic flies expressing 30 to 50 G4C2 repeat expansions, when compared to controls expressing 3–6 G4C2 repeats, displayed aberrant eye morphology, crawling and climbing defects, reduction of synaptic boutons and active zone at NMJs, and eclosion defect [127,264,265,266,267]. Flies expressing dipeptide repeat proteins were created by repeat associated non-ATG (RAN) translation that, by taking into account sense and antisense transcripts of G4C2 repeat expansions, resulted in five DRPs, that are poly-PA (prolinealanine), poly-PR (proline-arginine), poly-GR (glycine-arginine), poly-GA (glycinealanine), and poly-GP (glycine-proline) [268,269]. Several studies demonstrated that the expression of poly-GR or poly-PR caused severe eye degeneration and pupal lethality, whereas poly-GA, poly-GP, or poly-PA had no effect [265,270,271,272]. Nuclear localization of these DPRs provoked enlarged nuclei and nucleolar dysfunction [271,273]. Moreover, Tran and collaborators [274] have demonstrated that the expression of 160 G4C2 repeats did not induce toxicity through nuclear RNA foci but rather through cytoplasmic DPR proteins, suggesting that reducing repeat protein presence could be beneficial to ALS patients with *C9orf72* mutations.

## 8. Danio Rerio (Zebrafish) Models

Zebrafish (*Danio rerio*) is often used to study embryonic development because of the transparency of their embryos and their vertebrate body plan [275] and it is increasingly being used also in ALS [216,276,277,278]. Most human genes have a zebrafish homologue, typically with approximately 70% homology in protein sequence [275]. Thus, zebrafish can be used to study pathogenic mechanisms and disease progression as well as for drug screening [104,278,279]. In addition, gene overexpression or knockdown can be easily achieved by injections of RNA (or cDNA) or morpholinos (a form of antisense RNA) at the two- to four-cell embryonic stage. Gene-deletion strategies (such as CRISPR/Cas9) have also been used to contrast the off-target effects of some morpholinos [280]. Stable transgenic zebrafish can be used to study disease pathogenesis during ageing, with a lifespan of up to two years [281].

### 8.1. Zebrafish Carrying Cu/Zn SOD1 Mutations

As a first attempt to model ALS in zebrafish, human *SOD1*^G93A^, *SOD1*^G37R^, or *SOD1*^A4V^ mRNAs were injected into embryos resulting in abnormal motor neuron axon branching and shortened axon length. The latter mutation induced the most affected phenotype in a dose-dependent manner [282]. Similarly to SOD1 transgenic rodents, these phenotypes were more severe when expressing higher levels of mutant *SOD1*; whereas, disease features were absent when WT *SOD1* was overexpressed.

Subsequently, the *SOD1*^G93R^ and the *SOD1*^T70I^ zebrafish models have been developed [281,283]. With respect to current murine models, these transgenic zebrafishes offer the advantage of expressing the mutant *SOD1* at a physiological level, as occurs in ALS patients. All these *SOD1* variants induced key features of ALS, such as early NMJ alterations, susceptibility to oxidative stress, adult-onset muscular atrophy and paralysis, and premature death [284,285]. To conclude, these zebrafish ALS models are complementary to the existing mammal ones. Transgenic zebrafishes are often used in in-vitro and in-vivo studies for the development of new therapeutics [286,287,288].

### 8.2. Zebrafish Carrying TDP-43 Mutations

Zebrafish with the TDP-43^A315T^ mutation showed a marked decrease in motor axon length and increased aberrant branching with respect to controls expressing the human WT protein. Moreover, both WT and mutant TPD-43 proteins localized in the nucleus, thus indicating that cytoplasmatic translocation is not required to induce pathological defects [289]. Other models were obtained by introducing the G348C or the A382T mutation [290]. In the first case, zebrafish showed results almost superimposable to those of the A315T mouse mutants, whereas the A382T mutation only reduced axonal length. Nevertheless, all mutants manifested significant swimming impairment. Neurodegeneration and oxidative stress [291], locomotor deficiency, paralysis, and short lifespan also occurred [292,293]. Intriguingly, knocking down endogenous TDP-43 caused similar motor deficits and axonopathy, partly rescued by human wild type TDP-43 expression. This suggests the importance of TDP-43 functionality and that pathogenic mutations may cause both LoF and GoFc [290].

### 8.3. Zebrafish Carrying FUS Mutations

In the zebrafish model, both LoF and GoF of FUS function result in defective presynaptic function at the NMJ [294]. Expression of human R495X mutation in *FUS* resulted in the abrogation of a putative nuclear localization signal in zebrafish spinal cord and caused a striking cytoplasmic accumulation of the protein, somehow different from what observed for recessive (H517Q) and dominant (R521G) *FUS* mutants. Furthermore, the ALS-linked *FUS* mutants, but not the WT protein, assembled into perinuclear SGs in response to oxidative stress or heat shock conditions [295]. Moreover, in zebrafish expressing GFP-tagged WT or mutant R521C human *FUS*, mutant FUS mislocalized from the nucleus to the cytosol in cells other than MNs. Both WT and FUS^R521C^ localized at SGs, demonstrating an intrinsic propensity of human FUS to aggregate, independently of disease-associated mutations or specific cell type. However, elevation of the relative cytosolic to nuclear FUS induced by the R521C mutation led to a significant increase of SG assembly and persistence within vulnerable cells, although these cells were not always motor neurons [296]. *FUS* mutations also induced protein aggregation in MNs and other cells, oxidative stress, NMJ damage, and motor dysfunction [293,295,297,298,299]. As recently reported, deletion of the *FUS* orthologue in zebrafish led to homozygous mutants that displayed reduced lifespan and impaired motor abilities, associated with specific cellular deficits like decreased MN length and NMJ fragmentation. In addition, FUS LoF alters Tau transcripts, thus favoring the expression of small Tau isoforms [298,299].

### 8.4. Zebrafish Carrying C9orf72 Mutations

Both LoF and a GoF of C9orf72 have been investigated in the zebrafish model [278]. Deletion of the *C9orf72* sequence translated into altered neuronal development, MN axonopathy and axonal degeneration, disturbed arborization and shortened axons at early developmental stages, cytoplasmic aggregation of TDP-43, and abnormalities in spontaneous and evoked swimming. These deficits were rescued by expressing the human WT *C9orf72* mRNA, highlighting the specificity of the induced phenotype [300]. These data have been also confirmed by other groups [301,302], thus supporting that C9orf72 LoF mechanisms may underlie defects of the synaptic function at NMJ in ALS. On the other side, expression of longer repeats provokes C9orf72 GoF, which resulted in RNA foci initiating cell apoptosis [303], reduced motor axonal growth and aberrant branching [304]. A recent stable *C9orf72* transgenic zebrafish model, characterized by an accumulation of RNA foci and DPRs in muscle and in the central nervous system, showed motor defects and marked reduction of survival [305]. Additionally, muscle atrophy, loss of MNs, cognitive impairment, and early mortality also occurred in this model, which therefore, recaps many of the pathological hallmarks of ALS.

To reproduce the higher levels of DPRs observed in the CNS of *C9orf72* patients, zebrafish transgenic models have been obtained [269,306]. Lines with (GA80-GFP) or without (ggggcc80-GFP), an ATG codon forcing the translation of the poly-GA protein [307], were generated. Both lines presented RNA foci in neurons within the spinal cord. The expression of ggggcc80-GFP was only slightly toxic, while the expression of GA80-GFP manifested high toxicity, which was, however, rescued by an morpholino antisense able to interfere with GA80-GFP translation, thus suggesting that DPR reduction could represent a valuable therapeutic approach for ALS patients with *C9orf72* mutations. Additionally, the GA80-GFP model showed pericardial edema, reduced red blood cells, and muscle specific aggregates of GA80-GFP, but no significant differences in axon length and vascular pattern defects. Based on DPR toxicity, a stable transgenic zebrafish model expressing arginine-containing DPRs (poly-GR) has been developed [308]. In detail, ubiquitous expression of GR in zebrafish resulted in severe morphological and motor deficits, while selective GR expression in MNs provoked significant motors deficits without evident morphological alterations. In addition, decrease of MN axon length and increased cell apoptosis were observed in the spinal cord of zebrafish expressing GR specifically in MNs, while MN development did not seem to be affected.

## 9. Caenorhabditis Elegans Models

The nematode *Caenorhabditis elegans (C. elegans)* is another widely used ALS animal model [216,309]. Transgenic nematodes, with genes encoding for normal or disease-linked protein variants under neuronal specific promoters, are useful models to limit expression of mutated proteins to selected neuronal subtypes. The anatomical transparency of *C. elegans* makes the use of co-expressed fluorescent proteins useful to easily visualize neurons and monitor disease progression over time. Moreover, the well-defined and genetically manipulable nervous system of *C. elegans* provides an effective model to explore the pathological mechanisms of neurodegenerative diseases and a good tool for the screening of new potential drugs [310,311]. *C. elegans* also shares many conserved molecular pathways and cellular mechanisms with mammals, thus representing a reliable experimental model [312,313].

### 9.1. C. elegans Carrying SOD1 Mutations

In *C. elegans* models, neuronal expression of human *SOD1*^G85R^, but not WT SOD1 caused locomotor defects, cytosolic aggregates, axonal abnormalities, such as reduction in the number and diameter of cellular processes, and reduced numbers of organelles, both mitochondria and vesicles [314]. Nevertheless, no frank neuronal death was observed despite general functions were found to be affected, including survival, brood size, and rate of development survival. *C. elegans* expressing human *SOD1*^H46R^ or *SOD1*^H48Q^ also displayed locomotor defects, although to a lesser extent than *SOD1*^G85R^ [314]. Overexpression of human *SOD1*^G93A^ specifically in MNs led to age-dependent paralysis because of axonal defects [315]. Additionally, single-copy/knock-in models with A4V, H71Y, L84V, G85R, or G93A mutations of *SOD1* manifested differential toxicity toward glutamatergic and cholinergic neurons [316]. In particular, A4V, H71Y, G85R, and G93A mutants showed enhanced accumulation of SOD1 inclusion in cholinergic MNs, which were also more sensitive to paraquat-induced oxidative stress, possibly due to a toxic GoF. Conversely, oxidative stress-induced degeneration of glutamatergic neurons, which was observed in H71Y, L84V, and G85R mutants, was attributed to SOD1 LoF.

Interestingly, the specific overexpression of human *SOD1*, carrying the G85R, G93A, or G127X mutation in *C. elegans* muscles, formed different SOD1 aggregates according to the variant type, causing only mild cell dysfunction and reduced motility [317].

### 9.2. C. elegans Carrying TDP-43 Mutations

Phenotypic consequences of TDP-43 expression within *C. elegans* neurons have also been studied. Pan-neuronal expression of human wild-type TDP-43 in transgenic worms caused slowed and uncoordinated movements, as well as defasciculation of MNs [318]. On the other side, the expression of mutant variants of TDP-43, such as G290A, A315T, or M337V, caused the formation of nuclear TDP-43 insoluble aggregates and the specific neurodegeneration of GABAergic MNs that were accompanied by marked motility defects and progressive paralysis, leading to reduced lifespan [319]. In this invertebrate model, an ortholog of *TARDBP*, named *TDP-1*, has been identified in the nuclei of neurons and in the body wall muscle cells [320,321]. Interestingly, functional defects, like slower growth and altered locomotion, were induced in TDP-1 LoF mutants, and could be rescued by wild-type human TDP-43 expression [321]. Other studies showed that worms with *TDP-1* gene deletion did not manifest development alterations or significant movement impairment, and showed normal GABAergic synapses [318]. This is an opposite outcome with respect to mice and flies, suggesting a different role of *TDP-43* and *TDP-1* during evolution.

### 9.3. C. elegans Carrying FUS Mutations

It has been demonstrated that FUS variant expression, which induced protein aggregation specifically in GABAergic neurons, resulted in neurodegeneration, synaptic dysfunctions, and paralysis, whereas expression of wild-type FUS did not cause significant alterations [322].

Moreover, the pan-neuronal expression of two mutant *FUS* genes with increasing degree of clinical severity in ALS patients as to onset age and disease duration (P525L > R522G) resulted in proportional FUS cytoplasmic aggregation and impaired motor functions that progressed with age, associated to decreased lifespan. Conversely, animals expressing two *FUS* mutations associated with rather mild forms of human ALS (R514G, R521G) did not show protein aggregation and manifested no obvious dysfunctions compared with WT-*FUS* controls, suggesting that mutations give FUS a neurotoxic GoF [323].

### 9.4. C. elegans Carrying Deletion or Overexpression of C9orf72

Both C9otf72 LoF and GoF have been investigated in C. elegans models. The deletion of the *C9orf72* ortholog *alfa-1* resulted in altered nuclear transport, MN degeneration, and severe paralysis in early adulthood [324,325]. In addition, *alfa-1* deletion also caused the formation of worms with defects in lysosomal homeostasis, including dysfunctions in lysosomal reformation and the degradation of endocytosed elements, which were partially rescued by the expression of human WT *C9orf72* [325]. On the other side, *C. elegans* overexpressing 29 G4C2 repeats under the broadly active hsp-16 promoter displayed a more severe phenotype, including paralysis and death, compared to *C. elegans* overexpressing nine repeats only [326].

## 10. Saccharomyces Cerevisiae Models

It is well known that key cellular processes are conserved throughout yeast, higher eukaryotes, and humans and, indeed, yeasts like *Saccharomyces cerevisiae* can be used to study molecular pathways under both physiological and pathological conditions since approximately 6000 human genes [327] and 500 human disease genes have yeast orthologs [328]. In general, there are many advantages when working with yeast as it is easy to grow, preserve and store in the lab; it has a fully sequenced genome that can be easily manipulated and, as said above, it shares with human a high number of normal and disease-related gene families [329]. To date, the experimental approach to model diseases in yeast generally involves the overexpression of specific proteins and the monitoring whether their aggregation and localization alter cellular processes, possibly reducing cell viability. Of course, yeast does not recapitulate the specialized cell–cell interaction networks that occur in neural tissue but, due to the highly conserved eukaryotic machinery such as the protein quality control systems, it can be profitable to investigate alteration of protein processing, as a main feature of human neurodegenerative disorders [330]. In the last decade, yeast therefore emerged as a tool to investigate the molecular basis of several human neurodegenerative diseases that, like ALS, are characterized by protein misfolding and aggregation [331,332,333].

As a matter of fact, different studies have been performed using *Saccharomyces cerevisiae* carrying human WT or ALS-linked mutated genes for proteins such as SOD1, TDP-43, and FUS (for a recent and exhaustive review, see [330]).

### 10.1. Saccharomyces Cerevisiae Carrying SOD1 Mutations

One of the first attempts to model ALS in yeast was carried out by expressing WT or mutant (A4V, G39A, G93C, L38V) *SOD1* human genes in *Saccharomyces cerevisiae* lacking the ortholog gene [331]. However, expression of human mutants induced the restoration of SOD1 activity and resumed the WT phenotype, resistant to hyperoxic conditions (100% O_2_), and showed resistance to paraquat-induced oxidative stress. This study pointed out that many *SOD1* mutants displayed normal dismutasic activity, as supported also by studies on transgenic mice overexpressing human *SOD1* mutants [82,334,335]. Moreover, specific targeting of SOD1 into the yeast mitochondrial intermembrane space was shown to be protective against respiration-derived oxidative stress, arguing for the presence of functional SOD1 in this compartment [336,337]. To obtain a new SOD1 yeast model, different ALS-linked mutations (A4V, G37R, H48Q, G93A, and S134N) have been incorporated into the yeast *SOD1* gene [338]. The obtained data demonstrated that the mutant SOD1 isoforms are unstable, reducing cell viability without forming insoluble protein aggregates. In addition, such toxic effect does not seem to depend on mitochondrial dysfunction or oxidative stress, but rather on the inability to control central metabolic processes, most probably because of severe disruption of the vacuolar compartment.

### 10.2. Saccharomyces Cerevisiae Carrying TDP-43 Mutations

Unfortunately, yeast do not seem to possess *TARDP* orthologous genes. However, there are useful methods and approaches to gain insight into TDP-43 biology and its role in disease by using yeast [339].

The first yeast model with human WT TDP-43 overexpression showed that the protein accumulated in the cytoplasm and formed subcellular aggregates, which were found responsible for cell growth inhibition, deranged morphology, and cytotoxicity, thus recapitulating most of the features reported in cell culture models [340]. Interestingly, ALS-linked TDP-43 mutations (Q331K, M337V, Q343R, N345K, R361S, N390D) accelerated protein aggregation and increased the number of its cytosolic aggregates, leading to growth arrest and cell death [341]. In addition, two studies revealed that the depletion of the *Dbr1* gene coding for debranching enzyme in TDP-43 yeast model, strongly suppressed TDP-43-induced toxicity, impeding its interference with cellular RNAs and RNA-binding proteins [339,342]. Very recently, it has also been shown that deletion of *CNC1* (Cyclin C), *DNM1* (Dynamin-related), or *YBH3* (Bax inhibitor) genes, all involved in the oxidative stress-induced mitochondrial fragmentation, significantly reduced TDP-43-induced cell death in yeast [343].

### 10.3. Saccharomyces Cerevisiae Carrying FUS Mutations

As for TDP-43, yeast do not encode an *FUS* ortholog and, therefore, models have been induced by ectopically expressing human WT and/or mutated *FUS* genes in yeast [328,344,345,346]. These models have revealed a FUS GoF that is tightly associated with the degree to which the protein, following nuclear-to-cytoplasmic translocation, forms aggregates that co-localize with P-bodies and stress granules in the yeast cytoplasm [328,345,346,347] and inhibit the ubiquitin-proteasome system [348].

Removing the RNA recognition motif of FUS does not act on aggregates production but rescues FUS-induced toxicity, thus highlighting that the interaction between FUS and RNA is essential for FUS toxicity [346]. Consistently, recent studies in yeast identified several human genes able to suppress FUS toxicity, many of which were reported to express RNA-binding proteins [349].

## 11. Other Animal Models of ALS

Although mouse is still the most used model to investigate ALS and other neurodegenerative diseases, there are other mammals that have been used for translational studies on this dramatic disease. However, their use is more limited for logistic, economic, and ethical reasons.

### 11.1. Canine Models

Canine degenerative myelopathy (CDM) has been recognized as a spontaneous pathology occurring in specific dog strain, characterized by an adult-onset neurodegenerative spinal cord disorder and progressive impairment of motor functions [350,351]. CDM shares many molecular and clinical features with some forms of human ALS, such as the progression of the disease and the distribution of lesions like those reported for the upper MN-dominant onset form of ALS [352,353]. Thus, CDM has been considered an ALS model. To date, only two missense mutations in SOD1 dismutase, namely T18S and E40K, have been identified as the molecular determinants for CDM, showing recessive inheritance with reduced penetrance [354,355]. Both T18S and E40K mutations induced SOD1 aggregation possibly by reducing negative charge repulsion, by disrupting the E40-K41 salt bridge or by forming disulfide-linked enzymatically active dimers, thus supporting a SOD1 toxic GoF [356,357]. Besides MNs loss, canine models affected by CDM share some other pathological features with SOD1-ALS rodent models and patients, such as oligodendrocyte injury, leading to demyelination [358], increase of arginase 1-expressing microglia in the proximity of MNs [359], and upregulation of CB2 receptors in reactive astrocytes [360].

### 11.2. Swine Models

Swine models have acquired an emerging role due to their anatomical, physiological, and biochemical features closely related to the human ones, including genoma [361], model symptoms, and neuropsychiatric disease features [362]. Moreover, several neurodegenerative diseases have been modelled in swine [363,364,365,366,367,368], including ALS [369].

Chieppa and colleagues [369] produced the first G93A h*SOD1*-expressing swine model of ALS. Tg *SOD1* swine showed hind limb motor defects, which are germline transmissible, MN degeneration, in a mutated *SOD1* copies expression and age-dependent manner, gliosis and protein aggregates [370,371]. The disease onset occurred around 27 months of age, following a long presymptomatic phase characterized by increasing amounts of TDP-43 in peripheral blood mononuclear cells. Severe skeletal muscle pathology, including inflammation and necrosis, was observed mostly at the end stage of the disease [371]. In the early disease stage, however, mutant h*SOD1* does not form cytoplasmic inclusions, whereas nuclear accumulation and ubiquitinated nuclear aggregates are present, like in some ALS patient brains [370].

Later, Wang and colleagues [372] generated the first transgenic swine expressing mutant M337V TDP-43 and showing a severe phenotype and early death. At the molecular level, TDP-43 was detected in the cytoplasm of spinal cord and brain neurons, where it can interact with a protein-associated RNA splicing factor that associates with NeuN, thus altering neuronal RNA splicing, as also reported in ALS patients.

Notably, the utilization of large animal models like swine is useful in efficacy and/or safety studies of developing drugs as well as in gene silencing approaches using virally delivered shRNA, before their application in humans [367,373].

### 11.3. Non-Human Primate Models

Non-human primates (NHPs) are the animal models closest to humans with regards to genetics, physiology, and behavior. Therefore, they play a critical role in several biomedical and translational research projects, being the best species to model many human diseases [374], including neurodegenerative disorders [375,376].

A TDP-43-overexpressing *Macaca fascicularis* was created by injecting an AAV-based human WT TDP-43 coding sequence into the C5-6 spinal cord segment, ipsilaterally to the dominant hand [377]. After two to three weeks, monkeys manifested progressive motor weakness and muscle atrophy with fasciculation in the forelimb ipsilateral to the injected side; complete paralysis of the ipsilateral hand was observed two to five weeks after the onset. At the same time, muscle atrophy and weakness were also present on the contralateral side. At the cellular level, a diffuse TDP-43 mislocalization in the cytoplasm was evident in neurons, particularly α-MNs, but aggregates were not very frequent, indicating that this model indeed recapitulates the human in the spinal cord.

Besides their use to model ALS, monkeys represent also an unvaluable model to study safety and feasibility of novel therapeutic approaches before clinical experimentation. As a matter of fact, two studies have been carried out to test the safety and tolerability of the anti-SOD1 therapy in non-human primate. In the first case, was applied a silencing method based on an artificial microRNA (miR-SOD1), systemically delivered in the marmoset, by lumbar intrathecal administration of a recombinant adeno-associated virus (rAAVrh10; [378]). Using this construct, the silencing strategy significantly and safely resulted in ubiquitous marked reduction (65–93%) of SOD1 levels in lower motor neurons of the spinal cord. In the second study, the same approach was applied to macaques using rAAVrh10-miR-SOD1 with different constructs [379]. The procedure was well tolerated, and no significant adverse side effects were observed. Biodistribution analysis revealed widespread transduction in the spinal cord, in various brain areas, and in peripheral organs and was accompanied by a marked silencing of *SOD1* in spinal cord MNs. Recently, non-human primates have been used to test the potency, tolerability, and safety of single subpial AAV9 injection, demonstrating that AAV9 homogeneously distributes throughout the white and gray matter of cervical spinal cord and brain motor centers. This approach could be effective for subpial delivery of AAV9–shRNA–SOD1 to ameliorate clinical symptoms of ALS [373].

## 12. ALS-Related Protein Mutations for Novel or Potential Animal Models

Since 2014, new genes have been associated to ALS: *MATR3* [380], *CHCHD10* [381], *TBK1* [382,383], *TUBA4A* [384], *NEK1* [385,386], *ANXA11* [387], *C21orf2* [388], and *CCNF* [389]. Some of them have been translated into novel animal models; others are at present an interesting possibility, only.

### 12.1. MATR3 Mutations

Matrin 3 (MATR3) is a highly conserved RNA binding protein [390]. Three mutations of this protein have been identified (F115C, Y622A, P154S, and S85C) to be involved in both familial and sporadic ALS [380,391]. A transgenic mouse carrying the human WT *MATR3* was generated and developed either hindlimb paralysis or hindlimb and forelimb muscle atrophy [392]. Later, the same group, Moloney and collaborators [393], generated a transgenic *MATR3*^F115C^ mouse, showing myopathic changes that progressed from small vacuoles to large vacuolated fibers, rounded fibers, and fibers with internalized nuclei. Moreover, the *MATR3*^F115C^ mice developed muscle weakness with variable onset. Recently, a transgenic mouse that overexpresses mutant *MATR3*^S85C^ have been produced [394]. Overexpression of both WT or mutant *MATR3* caused myotoxicity. Very recently, a *MATR3*^S85C^ germline knock-in mouse was also generated [395]. Two independent groups found that MATR3 expression in *Drosophila* results in shortened lifespan and motor deficits, with disease-associated mutants exhibiting increased toxicity over MATR3(WT) [396,397]. Phenotype defects caused by muscle-specific *MATR3* expression were accentuated by the S85C pathogenic mutations.

### 12.2. CHCHD10 Mutations

Several mutations in the gene encoding the Coiled-Coil-Helix-Coiled-Coil-Helix Domain Containing 10 protein (*CHCHD10*) have been identified in families with ALS or ALS-FTD [381,398]. *CHCHD10* KO animals exhibited a slight pathological phenotype, with no bioenergetic defects or ultrastructural mitochondrial abnormalities in the brain, heart, or skeletal muscle [399]. Later, transgenic mice carrying the S59L mutation, were generated [400]. Eterozygous *CHCHD10^S59L^* mice displayed NMJ and MN degeneration, with fragmentation of the motor end plate and moderate motor neuron loss in lumbar spinal cord at the end stage of the disease, TDP-43 cytoplasmic aggregates in spinal neurons, and Mitochondrial Oxidative Phosphorylation System (OXPHOS) deficiency in muscle. *CHCHD19*^S55L^ knock-in mice were developed by two other independent groups [400,401]. Recently, Ryan and collaborators [402] generated transgenic mice overexpressing *CHCHD10*^R15L^, which showed transgene copy-linked abbreviated lifespan compared with mice over expressing WT *CHCHD10*. However, *CHCHD10*^R15L^ mice performed comparably to control mice in motor behavioral tasks, without developing paralysis.

### 12.3. TBK1 Mutations

Several TANK-binding kinase 1 (TBK1) mutations have been associated to ALS [382,383,403]. The association between ALS/FTD and TBK1 is almost exclusively based on loss-of-function mutations [404,405]. While homozygous loss of *TBK1* is embryonically lethal in mice, loss of one *TBK1* allele mirrors the genetic defect causing ALS/FTD in humans. [406]. In contrast, mice carrying both *TBK1* mutated allele, encoding a protein with no catalytic activity, were viable but developed severe immune cell infiltrates in multiple organs [407]. Furthermore, mice heterozygous knock-in for *TBK1*^G217R^, as well as homozygous and heterozygous mice expressing *TBK1*^R228H^, did not develop clinical or histological signs of motor symptoms up to two years of age [408]. Very recently, double mutant mice with both a heterozygous *TBK1* deletion and expression of human TDP-43^G298S^ have been generated [409]. Deletion of *TBK1* did not change the expression or cellular distribution of TDP-43 and did not provoke MN loss or reactive gliosis in the spinal cord. However, it caused muscle denervation and motor dysfunction.

### 12.4. CCNF Mutations

Mutations of cyclin F (CCNF) have been also recently correlated to ALS cases [389]. The first animal *CCNF* model was developed in zebrafish, based on an ALS-linked missense mutation [410]. This model exhibited cell death in the spinal cord, motor neuron axonopathy, consisting of shortened primary motor axons and increased frequency of aberrant axonal branching.

### 12.5. Other ALS-Related Mutations

Variants of the Tubulin Alpha 4A protein (TUBA4A) have been associated to ALS, with destabilization of the microtubule network and diminished repolymerization capability [384,411]. *TUBA4A* gene and protein expression dramatically increases with aging [384,412], while decreased levels of TUBA4A-mRNA have been found in the brain and spinal cord of sALS and fALS patients with mutations in *SOD1* and *C9orf72* [413]. No animal models are available at present; however, a neuron-like cell line with transient overexpression of ALS-related mutated forms of *TUBA4A* (R320C and A383T) showed altered neurite length and microtubule defects after exposure to selenium [414].

A significant association between the deletion of *NEK1* variants or the expression of *NEK1^R261H^* and FALS risk has been identified [385,386]. To date, animal models harboring ALS mutations in the *NEK1* gene are not available.

Mutations in the Annexin A11 (*ANXA11)* gene has been correlated to ALS, even if they are rare [387,415]. To date, animal models harboring ALS mutations in the *ANXA11* gene are not available.

Mutations in *C21orf2* have been recently related to ALS [388]. Bioinformatics and molecular modelling approaches revealed that both native and mutant structures of this protein might be deleterious, indicating that mouse would make a viable animal model to study this ALS gene in detail [416]. To date, very little evidence in the literature is present and no animal models are available.

## 13. Translational Burdens and Usefulness of In Vivo ALS Models

Although more than 50 disease-modifying drugs with different mechanisms of action have been studied in the last decades, only two have been approved for ALS patients, namely riluzole and edaravone that, however, have only very modest effects on the course of the disease [417,418]. Thus, there is an urgent need of effective therapies; unfortunately, this goal is hampered by the lack of the complete understanding of neuronal and non-neuronal mechanisms of MN degeneration [419].

Following the continuous identification of mutated genes, many animal models have been developed to unravel the pathological mechanisms that are crucial to MN degeneration. Indeed, the cellular alterations in ALS are likely the result of many different interacting mechanisms leading to a larger network disruption. This has been clearly exemplified in experimental models where multiple factors support neuronal damage [60]. The relative contribution of each of these factors to the human pathology cannot be fully ascertained; nonetheless, each of them should be considered in detail, as they represent the basis for current and future therapeutic perspectives. Unfortunately, most of the drugs tested so far in the preclinical studies and clinical trials were designed to counteract a single ALS causative factor with a constant high rate of failure. Moreover, preclinical data are usually derived from the analyses performed in a single animal model, making more difficult the translation into successful clinical trials that enroll non-stratified ALS patients, characterized by familial ALS, with different gene mutations, and sporadic ALS.

Modeling ALS as human neurodegenerative disorder into any other species, especially in mammals, is certainly a hard task in terms of face, construct, and predictive validity [420], and ALS is no exception.

Face validity regards whether the model recapitulates the main features of the pathology and its progression from both the clinical and anatomo-pathology point of view. Construct validity refers to what extent the cause of the experimentally-induced pathology reflects what causes the disease in patients. Finally, predictive value is the measure of the translational potential of the model, that is to what extent it predicts outcomes in patients, especially in terms of evaluation of therapeutic treatments.

There are no doubts that the models here summarized have been playing a key role in unravelling the myriad of cellular and molecular determinants that are involved in ALS and its progression, and in showing the multifactorial and non-cell autonomous nature of this disease. With regards to mammal models, especially rodents, it is evident that none fully recapitulate the characteristics of the human disease, but they reproduce most of the salient neuropathological and clinical features that are observed in ALS. Moreover, these models are all based on pathology-inducing genetic mutations, thus obviously having higher construct validity for familial than for sporadic ALS, but it is worth recalling that the two forms show common pathological mechanisms, share most neuropathological/clinical hallmarks, and up to 10% of sALS cases include fALS-associated gene mutations [421]. As for the predictive potential, interspecies and intraspecies variation could certainly play a major role in complicating the interpretation of the results and making their translation to the clinic not so straightforward. As a matter of fact, the very many therapies showing beneficial effects in animal studies failed to significantly impact the disease progression in humans. These failures may be due to the heterogeneity of human ALS with respect to the more homogeneous phenotype in animals. This is obviously true and an improved selection of patients during recruitment, based on biomarker availability, will surely abate this drawback. However, this pitfall partially represents the reason of the mismatch between animal and human studies, being the accurate management of animal experimentations the other important side of the coin.

Similarly to human studies, accurate preclinical guidelines for phase-based efficacy evaluation of innovative therapeutic approaches and more transparency in reporting the experiments involving animals [422,423] would produce more reliable results than the majority of the current studies present in the literature. Indeed, the rationale for the choice of a specific ALS model, experimental design details, and access to the data obtained can be very useful to better interpret the results, particularly by clinicians who try to translate into clinical trials the most promising preclinical data.

## 14. Conclusions

**Figure 1:**
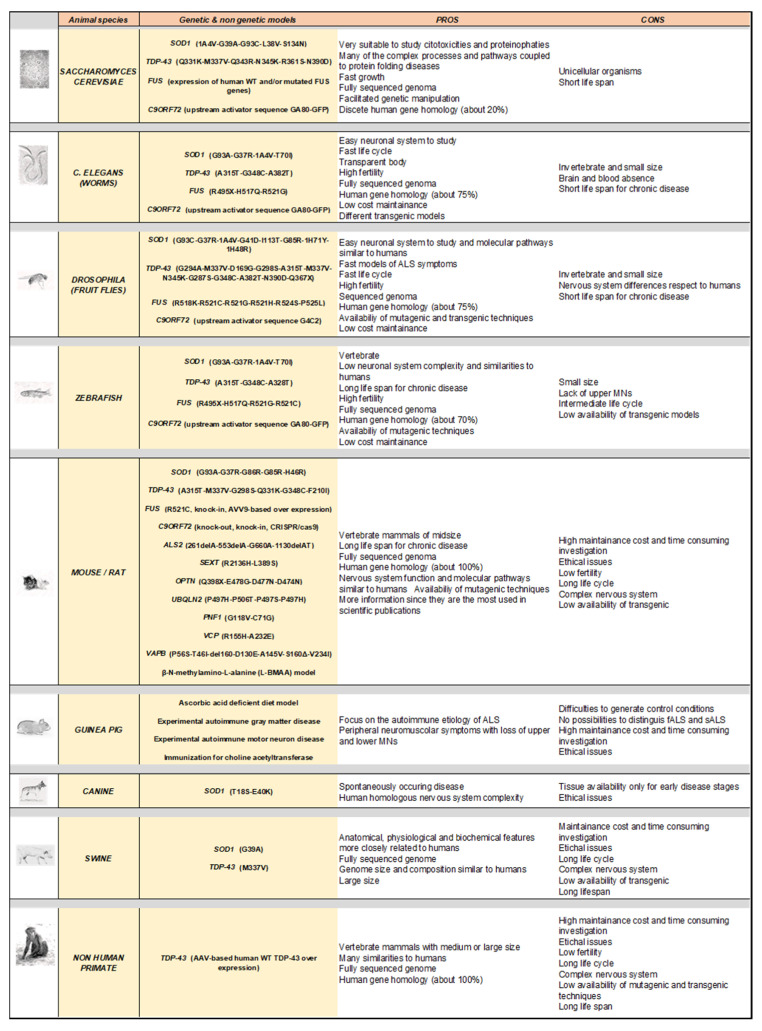
Pros and cons of available ALS animal models.

In this review, we provide an overview of the different experimental models, from yeast to non-human primates, to investigate ALS pathophysiology. We clustered these models on a genetic mutation criterion, highlighting the peculiarities and the ALS-related molecular, cellular, and clinical symptoms, also focusing on pros and cons that need to be considered when approaching the study of ALS pathogenesis or new experimental therapies (Figure 1). Clearly, the main advantage of in vivo over in vitro studies is the higher chance to clarify the multifaced pathological mechanisms that characterize the human pathology during disease progression. Although a comparative analysis of these models is required to choose the best one(s) required for the planned investigation, if we want to better understand the causes of ALS, we should never forget their limitations.

## Data Availability

Not Applicable.
